# Elevated serum tartrate-resistant acid phosphatase isoform 5a levels in metabolic syndrome

**DOI:** 10.18632/oncotarget.17839

**Published:** 2017-05-12

**Authors:** Yi-Jhih Huang, Tsai-Wang Huang, Tsu-Yi Chao, Yu-Shan Sun, Shyi-Jou Chen, Der-Ming Chu, Wei-Liang Chen, Li-Wei Wu

**Affiliations:** ^1^ Division of Thoracic Surgery, Department of Surgery, Tri-Service General Hospital, National Defense Medical Center, Taipei, Taiwan, Republic of China (R.O.C); ^2^ Division of Oncology, Department of Internal Medicine, Tri-Service General Hospital, National Defense Medical Center, Taipei, Taiwan, Republic of China (R.O.C); ^3^ Shuang Ho Hospital, Taipei Medical University, Taipei, Taiwan, Republic of China (R.O.C); ^4^ Division of Family Medicine, Tri-Service General Hospital Penghu Branch, National Defense Medical Center, Taipei, Taiwan, Republic of China (R.O.C); ^5^ Department of Pediatrics, Tri-Service General Hospital, National Defense Medical Center, Taipei, Taiwan, Republic of China (R.O.C); ^6^ Division of Family Medicine, Department of Family and Community Medicine, Tri-Service General Hospital, Taipei, Taiwan, Republic of China (R.O.C); ^7^ Division of Geriatric Medicine, Department of Family and Community Medicine, Tri-Service General Hospital, Taipei, Taiwan, Republic of China (R.O.C); ^8^ Graduate Institute of Medical Sciences, National Defense Medical Center, Taipei, Taiwan, Republic of China (R.O.C)

**Keywords:** metabolic syndrome, tartrate-resistant acid phosphatase

## Abstract

**Background:**

Tartrate-resistant phosphatase isoform 5a is expressed in tumor-associated macrophages and is a biomarker of chronic inflammation. Herein, we correlated serum tartrate-resistant phosphatase isoform 5a levels with metabolic syndrome status and made comparisons with traditional markers of inflammation, including c-reactive protein and interleukin-6.

**Methods:**

One hundred healthy volunteers were randomly selected, and cut-off points for metabolic syndrome related inflammatory biomarkers were determined using receiver operating characteristic curves. Linear and logistic regression models were subsequently used to correlate inflammatory markers with the risk of metabolic syndrome.

**Results:**

Twenty-two participants met the criteria for metabolic syndrome, and serum tartrate-resistant phosphatase isoform 5a levels of >5.8 μg/L were associated with metabolic syndrome (c-statistics, 0.730; p = 0.001; 95% confidence interval, 0.618–0.842). In addition, 1 μg/L increases in tartrate-resistant phosphatase isoform 5a levels were indicative of a 1.860 fold increase in the risk of metabolic syndrome (p = 0.012).

**Conclusions:**

Elevated serum tartrate-resistant phosphatase isoform 5a levels are associated with the risk of metabolic syndrome, with a cut-off level of 5.8 μg/L.

## INTRODUCTION

Tartrate-resistant acid phosphatase (TRACP) isoforms 5a and 5b are expressed in monocytes during inflammation, and TRACP-5b is a biomarker for osteoclast activity and has been correlated with osteolytic responses, bone resorption, and bony metastases [1–5]. In contrast, TRACP-5a has been identified as a biomarker for cancer cachexia, metastatic breast cancer progression, end-stage renal disease, rheumatoid arthritis, and sarcoidosis [6–10] and as a potential risk factor for cardiovascular disease [11].

Multiple biomarkers are under investigation for their correlations with cardiovascular complications of metabolic syndrome (MetS) [12–19]. Documented inflammatory markers include interleukin-6 (IL-6), high sensitivity C-reactive protein (CRP), procalcitonin, calprotectin, and osteocalcin [12, 13, 15, 17, 19, 20]. Among these, CRP levels are strongly associated with cardiovascular risk [13, 19, 21, 22], but are sensitive to infection and various clinical and subclinical inflammatory reactions. In contrast, TRACP-5a may be a more specific biomarker for chronic inflammatory responses associated with MetS [8–11].

MetS is an aggregate of cardiovascular risk factors, including hypertension, obesity, dyslipidemia, and blood glucose intolerance [21, 23–26]. Patients with MetS are defined by the presence of at least three of the following characteristics based on the revised National Cholesterol Education Program's Adult Treatment Panel III [27]: (1) systolic blood pressure (SBP) ≥ 130 mmHg or diastolic blood pressure (DBP) ≥ 85 mmHg; (2) waist circumference ≥ 90 cm in males or ≥ 80 cm in females; (3) high-density lipoprotein (HDL) < 40 mg/dL (1.03 mmol/L) in males or < 50 mg/dl (1.29 mmol/L) in females; (4) fasting glucose ≥ 100 mg/dl (5.6 mmol/L); and (5) triglyceride levels ≥ 150 mg/dL (1.7 mmol/L). Recent studies have correlated cardiovascular events and MetS with chronic subclinical inflammatory responses [22, 28], potentially reflecting increased oxidative stress due to dyslipidemia and insulin resistance, and the ensuing monocyte-associated vascular endothelial damage. In this study, we investigated TRACP-5a as an independent risk factor for MetS.

## RESULTS

### Demographic characteristics

Demographic characteristics of the 100 participants were stratified by sex and are presented in Table 1. The male to female ratio was 88:12, and twenty-two participants met the criteria for MetS. Components and distributions of MetS between male and female subjects were similar, except that blood pressure was predominantly higher in the male group. Age, body mass index, fasting glucose levels, and lipid profiles did not differ significantly between male and female study groups (p > 0.05).

**Table 1:** Demographic characteristics and metabolic syndrome components of the 100 study participants

Variables, mean(SD)	Male (N = 88)	Female (N = 12)	P-value
MetS, n(%)	19(21.6)	3(25)	0.723*
Age	27.79(4.05)	26.97(2.27)	0.466
SBP	121.5(14.3)	110.9(9.80)	<0.05†
DBP	78.34(10.1)	70.08(8.32)	<0.05†
Body mass index	27.79(4.05)	26.97(2.27)	0.307
Waist circumference (cm)	87.36(10.1)	82.25(8.72)	0.081
Triglyceride (mg/dL)	114.8(58.5)	92.42(50.3)	0.177
High-density lipoprotein (mg/dL)	44.45(9.66)	52.08(14.5)	0.102
Fasting glucose (mg/dL)	94.25(10.5)	91.08(5.48)	0.116
IL-6 (pg/mL)	3.41(16.2)	1.40(3.51)	0.670
CRP (mg/dL)	1.53(1.68)	1.97(1.48)	0.385
TRACP-5a (μg/L)	6.63(2.38)	4.99(2.13)	0.026†

*Fisher's exact test, †p < 0.05.

SD, standard deviation; SBP, systolic blood pressure; DBP, diastolic blood pressure.

### C-statistics of MetS predictive inflammatory markers

**Figure 1:**
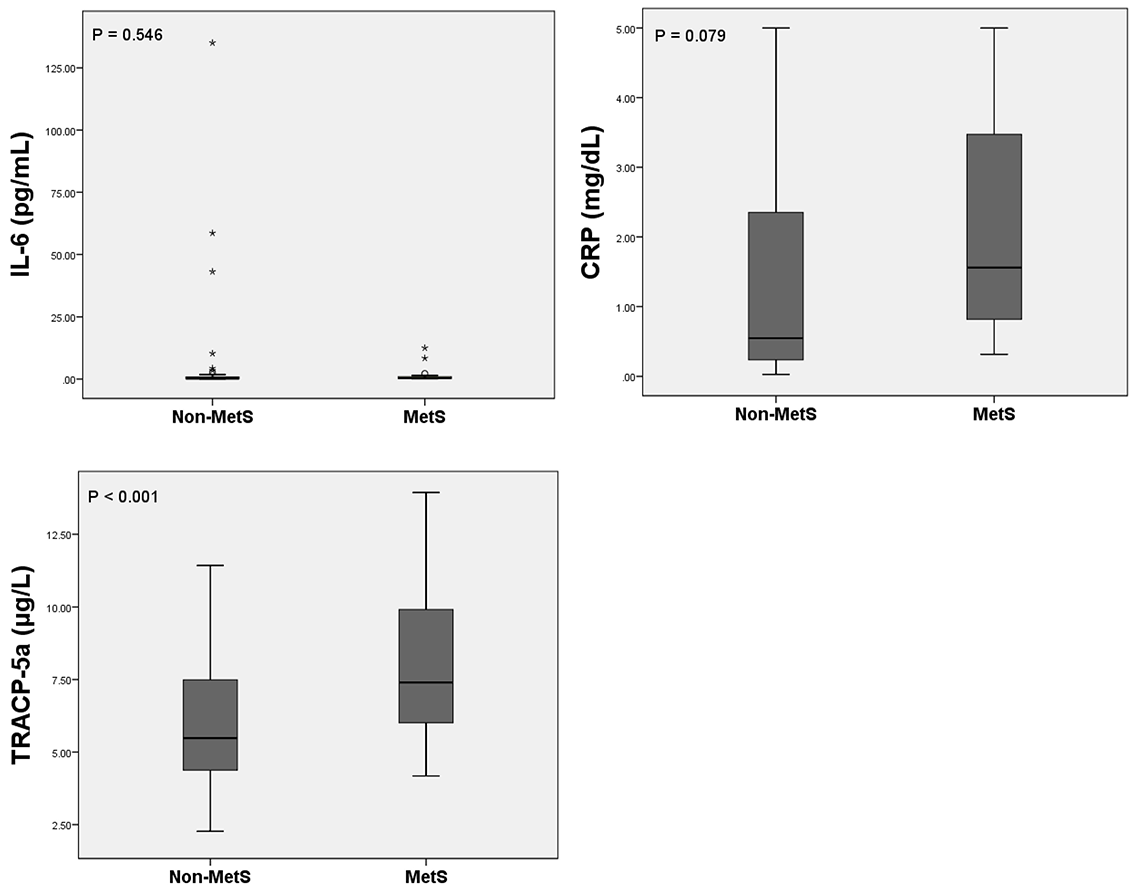
Receiver operating characteristic curves of inflammatory biomarkers of MetS IL-6, interleukin-6; TRACP-5a, tartrate-resistant acid phosphatase isoform 5a.

Ranges of IL-6, CRP, and TRACP-5a levels are presented in Table 2, and receiver operating characteristic curves are shown in Figure 1. After applying c-statistics, the area under the curve for TRACP-5a was 0.730 (p = 0.001, 95% CI, 0.618–0.842) and those of CRP and IL-6 were 0.693 (p = 0.006, 95% CI, 0.587–0.799) and 0.563 (p = 0.369, 95% CI, 0.430–0.696). The optimal cut-off point for TRACP-5a and CRP levels were 5.8005 μg/L and 0.7604 mg/dL, respectively.

**Table 2:** C-statistics for the risk of metabolic syndrome using inflammatory biomarkers

Inflammatory markers	Sensitivity	Specificity	Cut-off point	C-statistic (95% C.I.)	P-value
IL-6 (pg/mL)	0.682	0.474	0.2055	0.563 (0.430-0.696)	0.369
CRP (mg/dL)	0.864	0.590	0.7604	0.693 (0.587-0.799)	0.006†
TRACP-5a (μg/L)	0.864	0.551	5.8005	0.730 (0.618-0.842)	0.001†

†p < 0.01.

The best deterministic value is a C-statistic (area under curve) of 1.

IL-6, Interleukin-6; TRACP-5a, tartrate-resistant acid phosphatase isoform 5a; CRP, C-reactive protein; C.I., confidence interval.

### Comparison of MetS to non-MetS patients

**Figure 2:**
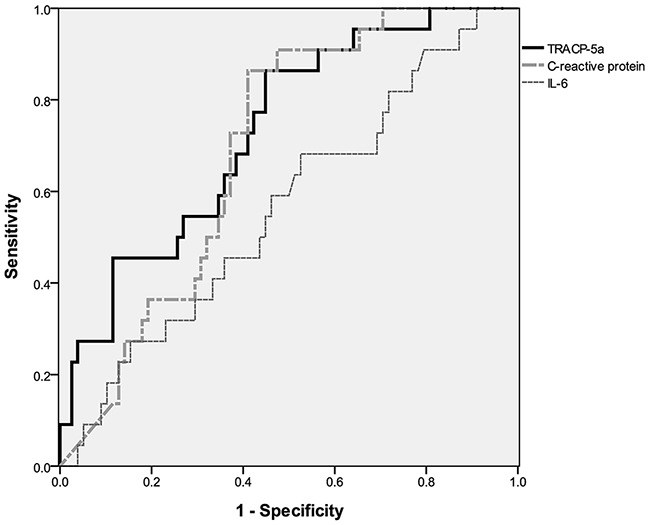
Serum TRACP-5a levels are significantly higher in participants with metabolic syndrome MetS, metabolic syndrome; IL-6, interleukin-6; CRP, C-reactive protein; TRACP-5a, tartrate-resistant acid phosphatase isoform 5a.

Although fasting glucose levels did not differ significantly between MetS and non-MetS subjects (p = 0.069), systolic blood pressure (p = 0.032), diastolic blood pressure (p = 0.001), waist circumference (p = 0.002), triglyceride levels (p < 0.001), and HDL levels (p < 0.001) differed significantly. IL-6 (p = 0.546) and CRP levels (p = 0.079) did not differ between patients with and without MetS, whereas TRACP-5a levels were significantly higher in subjects with MetS (p < 0.001; Figure 2, Table 3). No significant differences in blood urea nitrogen, creatinine, aspartate aminotransferase (AST), alanine aminotransferase (ALT), fasting glucose, and uric acid levels, or other blood cell parameters were identified between MetS and non-MetS patients.

**Table 3:** Comparison of the components of metabolic syndrome and TRACP-5a classificationsystems

Variables, mean (SD)	(A) Metabolic syndrome (MetS)			(B) TRACP-5a		
	Non-MetS	MetS	P-value	≤ 5.8(μg/L)	> 5.8(μg/L)	P-value
	N = 78	N = 22		N = 46	N = 54	
Metabolic syndrome, n (%)	-	-	N/A	3 (6.52%)	19 (35.2%)	<0.001‡
Age (years)	30.18 (6.85)	36.45 (7.46)	<0.001‡	30.63 (7.48)	32.35 (7.35)	0.250
Body height	172.7 (7.12)	170.9 (7.86)	0.308	171.8 (7.61)	172.7 (7.04)	0.522
Body weight (kg)	80.99 (12.0)	86.09 (12.5)	0.085	79.87 (12.4)	84.02 (11.9)	0.092
Systolic blood pressure (mmHg)	118.6 (13.7)	125.9 (14.6)	0.032*	116.7 (11.6)	123.2 (15.5)	0.021*
Diastolic blood pressure (mmHg)	75.56 (9.33)	83.68 (10.8)	0.001†	73.83 (8.69)	80.35 (10.5)	0.001†
Waist circumference	85.11 (9.88)	92.55 (8.46)	0.002†	84.39 (10.4)	88.75 (9.31)	0.030*
Serological exams						
Total cholesterol (mg/dL)	167.5 (30.1)	191.0 (28.7)	0.001†	166.0 (33.1)	178.4 (28.6)	0.047*
Triglyceride (mg/dL)	93.68 (38.7)	177.3 (67.3)	<0.001‡	93.28 (44.6)	128.1 (63.0)	0.002†
High-density lipoprotein (mg/dL)	47.22 (9.66)	38.80 (11.2)	<0.001‡	48.11 (11.6)	43.03 (9.05)	0.016*
Low-density lipoprotein (mg/dL)	108.1 (29.1)	128.0 (25.9)	0.005†	104.7 (30.1)	119.1 (27.4)	0.014*
Fasting glucose (mg/dL)	93.00 (9.01)	97.32 (12.8)	0.069	92.04 (9.80)	95.43 (10.1)	0.094
Inflammatory markers						
IL-6	3.659 (17.2)	1.425 (3.04)	0.546	1.583(8.59)	4.517(19.1)	0.339
CRP	1.429 (1.64)	2.130 (1.61)	0.079	1.336(1.53)	1.794(1.74)	0.169
TRACP-5a	5.986 (2.18)	8.013 (2.54)	<0.001‡	-	-	N/A

*p < 0.05, †p < 0.01, ‡p < 0.001.

TRACP-5a, tartrate-resistant acid phosphatase isoform 5a; SD, standard deviation; IL-6, interleukin-6; CRP, C-reactive protein.

In subsequent c-statistics analyzes, participants were classified in terms of MetS risk according to a TRACP-5a cut-off level of 5.8 μg/L. This cut-off point was accompanied by significant differences in AST and ALT levels, white blood cell counts, red blood cell counts, and hemoglobin levels. Moreover, blood pressure, waist circumference, and lipid profiles corresponded significantly with TRACP-5a levels.

### Elevated TRACP-5a was associated with increased risk of MetS

In further analyses, binary logistic regression models were used to adjust parameters between MetS and non-MetS groups and to determine the risk of MetS (Table 4). Based on the data in Table 3 (column A), we controlled factors that differed significantly between MetS and non-MetS groups. In regression model 1, age, total cholesterol, and low-density lipoprotein (p = 0.007, 95% CI, 1.097 to 1.809) were covariates, whereas regression model 2 included the inflammatory parameters in model 1 with SBP, DBP, waist circumference, triglyceride, HDL, and fasting glucose (p = 0.012, 95% CI, 1.148–3.013). In models 1 and 2, single unit increases in TRACP-5a levels correlated with 1.409- and 1.860-fold increases in the risk for MetS. In contrast, IL-6 and CRP levels were not predictive of the risk for MetS.

**Table 4:** Binary regression models of MetS predictive inflammatory markers

Variables	(A) Metabolic syndrome			
	Model 1		Model 2	
	OR (95% CI)	P-value	OR (95% CI)	P-value
IL-6	0.989 (0.935, 1.046)	0.697	0.997 (0.901, 1.103)	0.955
CRP	1.097 (0.787, 1.531)	0.584	0.984 (0.593, 1.635)	0.951
TRACP-5a	1.409 (1.097, 1.809)	0.007†	1.860 (1.148, 3.013)	0.012*

Variables of Table 3, column (A) that reached statistically significance were controlled by the binary regression model.

*p < 0.05, †p < 0.01.

Model 1, age + total cholesterol + low-density lipoprotein.

Model 2, Model 1 + the metabolic syndrome components systolic blood pressure, diastolic blood pressure, waist circumference, triglyceride, high-density lipoprotein, and fasting glucose.

IL-6, interleukin-6; CRP, C-reactive protein; TRACP-5a, tartrate-resistant acid phosphatase isoform 5a; CI, confidence interval; OR, odds ratio.

### Elevated blood sugar and triglyceride levels are associated with increments in TRACP-5a

We used linear regression models to identify MetS components that influence serum TRACP-5a concentrations (Table 5). In these analyzes, model 1 included sex only, model 2 included model 1 with AST, ALT, and uric acid levels, and model 3 comprised the parameters of model 2 with white and red blood cell counts and hemoglobin levels. After controlling for significant parameters from the TRACP-5a group (Table 3, column B), MetS components were not cumulatively associated with TRACP-5a levels until the total number of MetS components was ≥3 (Table 5), indicating that the presence of MetS is an independent predictor of TRACP-5a levels. However, further analyses of the effects of each component on TRACP-5a levels showed that impaired glucose tolerance and excess triglyceride levels are crucial determinants of TRACP-5a levels (p = 0.006 and p = 0.031, respectively).

**Table 5:** Linear regression analyzes of MetS components that are predictive of TRACP-5a levels (μg/L)

Variables	(B) TRACP-5a level					
	Model 1		Model 2		Model 3	
	β (95% CI)	P-value	β (95% CI)	P-value	β (95% CI)	P-value
Numbers of MetS components						
0	−0.859 (−2.096, 0.378)	0.171	−0.362 (−1.585, 0.862)	0.559	−0.265 (−1.501, 0.970)	0.671
1	−0.844 (−1.837, 0.149)	0.095	−0.641 (−1.586, 0.304)	0.181	−0.487 (−1.473, 0.500)	0.330
2	−0.239 (−1.273, 0.794)	0.647	−0.591 (−1.576, 0.395)	0.237	−0.876 (−1.879, 0.126)	0.086
≥3 (Metabolic syndrome)	2.062 (1.008, 3.116)	<0.001‡	1.821 (0.801, 2.841)	<0.001‡	1.771 (0.753, 2.788)	<0.001‡
Factors of metabolic syndrome						
Central obesity	−0.254 (−1.219, 0.710)	0.602	−0.395 (−1.320, 0.530)	0.399	−0.488 (−1.413, 0.437)	0.298
Low HDL level	0.404 (−0.591, 1.399)	0.422	0.429 (−0.535, 1.392)	0.379	0.274 (−0.692, 1.240)	0.574
Elevated blood pressure	1.284 (0.317, 2.250)	0.010†	1.108 (0.174, 2.042)	0.021*	0.938 (−0.010, 1.886)	0.052
High TG level	1.747 (0.669, 2.825)	0.002†	1.299 (0.226, 2.372)	0.018*	1.196 (0.115, 2.277)	0.031*
High blood sugar	2.017 (0.886, 3.149)	<0.001‡	1.520 (0.351, 2.689)	0.011*	1.660 (0.489, 2.831)	0.006†

The definition for the factors of metabolic syndrome is consistent with the revised National Cholesterol Education Program's Adult Treatment Panel III. Variables of Table 3, column (B) that reached statistically significance were adjusted by the linear regression model.

*p < 0.05, †p < 0.01, ‡p < 0.001.

Model 1, sex.

Model 2, Model 1 + (AST, ALT, uric acid).

Model 3, Model 2 + (white blood cell, red blood cell, hemoglobin).

HDL, high-density lipoprotein; TG, triglyceride; AST, aspartate aminotransferase; ALT, alanine aminotransferase.

## DISCUSSION

TRACP-5a is a monocyte-derived specific biomarker for chronic inflammatory responses and has been correlated with cardiovascular disease. TRACP-5a is also reported to be a biomarker for many benign or malignant diseases [6–10]. Chen et al. reported that the serum TRACP-5a level lower than 12.4 μg/L had better survival for metastatic breast cancer [7]. The serum TRACP-5a level also elevated significantly in active sarcoidosis compared with control group (11.66 versus 8.04 μg/L) [8]. Meanwhile, TRACP-5a levels were elevated in end-stage renal disease and rheumatoid arthritis in Janckila's study [9, 10].

However, circulating monocytes do not secrete TRACPs prior to differentiation into macrophages [11]. Accordingly, we hypothesized that serum TRACP-5a levels are associated with MetS, which is a cluster of cardiovascular risk factors. In this prospective study, we demonstrated a significant association between MetS and TRACP-5a levels and revealed the underlying mechanisms. Specifically, serum TRACP-5a levels were correlated with elevated blood pressure, hyperlipidemia, and insulin resistance, and the TRACP-5a biomarker cut-off value of 5.8 μg/L was defined as indicative of higher MetS risk using c-statistics.

Previous studies have shown that cardiovascular disease is initiated by endothelial damage and consequent release of cytokines. These molecules (1) induce circulating monocyte adherence and transformation into macrophages, (2) induce lipid ingestion and aggregation within foam cells, and (3) transform foam cells into atherosclerotic plaques [29]. Traditional risk factors for cardiovascular disease include sex, age, smoking history, hypertension, dyslipidemia, insulin resistance, and central obesity [30]. In addition, it is well documented that cardiovascular events and MetS are strongly associated with chronic inflammation [22, 31, 32].

In the present comparisons of MetS and non-MetS patient groups, TRACP-5a levels differed significantly, whereas IL-6 and CRP levels did not. In addition, among the present parameters, only age differed significantly between MetS and non-MetS groups. These data are in agreement with a previous cohort study of females showing that serum TRACP-5a levels increase with age [33]. However, age and TRACP-5a levels were not significantly correlated according to Pearson's coefficient (r = 0.192, p = 0.055), suggesting that increasing age is not strongly associated with differences in TRACP-5a levels between MetS and non-MetS patients.

AST, ALT, and uric acid levels, white and red blood cell counts, and hemoglobin levels were significantly stratified at the TRACP-5a cut-off of 5.8 μg/L (Table 3). However, in the present linear regression models of TRACP-5a levels, none of these parameters were significant independent correlates (p > 0.05 of β coefficient in each regression model). However, among patients that met at least three criteria for MetS, these variables were correlated with TRACP-5a levels (Table 5).

After controlling for other variables, elevated triglyceride and blood sugar were predictive of TRACP-5a levels. Insulin resistance has been associated with both serum IL-6 and CRP levels in previous studies [34–36], and concomitant improvements (reductions) in these parameters have been observed following exercise training for 12 weeks in obese subjects, whereas TRACP-5a titers remained elevated [37]. In contrast, TRACP-5a, CRP, and IL-6 levels were decreased with weight loss twelve months after bariatric surgery (gastric bypass surgery or vertical banded gastric partition) in severely obese subjects [38]. Taken with the present observations, these data suggest that TRACP-5a secretion during macrophage infiltration is associated with circulating triglyceride levels and is affected by incremental changes in adipose tissue volumes in vessel endothelia.

The present study was limited by sex bias, with male subjects comprising 80% of the cohort, although the proportion of males-to-females in MetS vs. non-MetS groups was equal (p = 0.519). This study was also limited by its cross-sectional design, which prevented evaluations of TRACP-5a responses to treatments such as lifestyle modification, exercise, and medication. Thus, future studies are required to confirm the present observations in larger cohorts of both sexes. Moreover, TRACP-5a accumulation in vascular endothelial cells needs to be verified and evaluated in autopsies from animal models. Finally, as the sample size was small, further studies are required to define relationships between TRACP-5a and individual MetS components, and the effects of treatments for insulin resistance and inflammatory markers.

In conclusion, this study warrants further consideration of increasing TRACP-5a levels as a more specific biomarker than the traditional MetS inflammatory IL-6 and CRP. Specifically, the present analyzes suggest that >5.8 μg/L TRACP-5a level is a potential cut-off point for the presence of MetS.

## MATERIALS AND METHODS

### Study design, participants, and blood sample collection

This was a preliminary study for observing the levels and trends of inflammatory markers in patients with MetS. This study was conducted with the annual physical examination for civilians who received physical check-up every year at a single institution. Subjects with a history of coronary artery disease or medication for hypertension, dyslipidemia, blood sugar control, or malignancy were excluded. We requested for volunteers who were willing to participate in our study without selection and the expected final participant number was designated as 100 in the beginning of this study. From April 2015 to June 2015, 100 healthy civilian volunteers were enrolled. All participants received physical examinations and serum biochemical and hematologic analyzes, and inflammatory biomarker evaluations were performed to determine IL-6, CRP, and TRACP-5a titers. Blood samples were collected in the morning after at least 8 hours (h) of fasting and were stored at −80°C within 30 min of sampling. The study was approved by the Institutional Review Board of Tri-Service General Hospital (TSGH-IRB approval number: 1-103-05-065). Written informed consent was obtained from each participant prior to participation in the study. All laboratory methods were performed in accordance with the related guidelines.

### Biochemical markers and serologic data

TRACP-5a titers were assessed using a two-site immunoassay as described previously [11]. Briefly, approximately 10 μL aliquots of serum were washed and diluted with 90 μL buffer (10 mM Tris-HCl, 150 mM NaCl, pH 7.5, containing 2% glycerol, 10 mM EDTA, and 0.05% Tween-20 solution) and were added in duplicate to 1 mg biotinylated mab220-coated streptavidin wells (Pierce Chemical Co.) that were specific for TRACP-5a. Diluted samples were covered with plastic wrap and were then incubated for 16 h (overnight) at 4°C. Streptavidin wells were then washed, and 100 μL aliquots of horseradish peroxidase-conjugated anti-TRACP mab162 diluted at 1:1000 were added and incubated for 1 h at room temperature. Streptavidin wells were washed again and o-phenylenediamine dihydrochloride and H_2_O_2_ (horseradish peroxidase substrate) at pH 5.0 was added. Wells were then mixed and incubated for precisely 15 min and reactions were stopped by adding 50 μL aliquots of 2 M H_2_SO_4_. CRP levels were analyzed using enzyme-linked sandwich immunoassays with rabbit polyclonal antiserum CRP (DAKO Denmark). Serum IL-6 titers were determined using a commercial immunoassay kit (RayBiotech, Inc.).

### Statistical analysis

Statistical analyzes were performed using Statistical Product and Service Solutions (SPSS) software (version 18.0; SPSS, Chicago, IL, USA). Descriptive data were expressed as means ± standard deviations and categorical variables were compared using chi-square or Fisher's exact tests. Differences between continuous variables and categories were identified using student's t-test, and c-statistics were used to determine cut-off points for inflammatory markers. Significant associations of parameters with MetS were identified using linear and binary regression analyzes. Differences and associations were considered significant when p < 0.05.

## Abbreviations

CI: Confidence interval
CRP: C-reactive protein
DBP: Diastolic blood pressure
HDL: High-density lipoprotein
MetS: Metabolic syndrome
R.O.C: Republic of China
SBP: Systolic blood pressure
SPSS: Statistical Product and Service Solutions
TRACP: Tartrate-resistant acid phosphatase
